# Associations between Global DNA Methylation and Telomere Length in Healthy Adolescents

**DOI:** 10.1038/s41598-017-04493-z

**Published:** 2017-06-23

**Authors:** Yutong Dong, Ying Huang, Bernard Gutin, Anas Raed, Yanbin Dong, Haidong Zhu

**Affiliations:** 1 Georgia Prevention Institute, Department of Pediatrics, Medical College of Georgia, Augusta University, Augusta, Georgia 30912 USA; 2 Internal Medicine, Department of Medicine, Medical College of Georgia, Augusta University, Augusta, Georgia 30912 USA

## Abstract

Emerging evidence suggests that epigenetics regulates telomere dynamics in adults. However, the relationship between these pathways in children and youth remains unknown. Thus, we examined this association in 542 healthy adolescents aged 14 to 18 years old (44.8% African Americans; 55.2% females). Global DNA methylation level (%5-mC) was quantified using ELISA method. Leukocyte telomere length (LTL) was defined as relative telomere to single copy gene (T/S) ratio. Multiple linear regression models, adjusted for age, gender, ethnicity, Tanner stage, BMI, PA, and batch effect, revealed that %5 mC was associated with LTL (adjusted β = 0.17, *p < *0.01). %5 mC accounted for 5.0% of the variation for LTL. A significant gender interaction was identified (*p < *0.01). There was an association between %5 mC and LTL in females (all *ps* < 0.01), but not in males. Further sensitivity analyses by race revealed similar associations in African Americans and whites (all *ps* < 0.03). The present study, for the first time, shows that lower levels of global DNA methylation are associated with shorter telomere lengths in youth, which may decrease genome stability and augment the susceptibility to diseases. Longitudinal studies are warranted to establish the effects of global DNA methylation on LTL maintenance over time.

## Introduction

Global DNA methylation is an important epigenetic mechanism, starting as early as fetal development, regulating gene expression, chromosomal stability, and disease susceptibility^1, 2^. The downregulation of DNA methylation levels in long interspersed nuclear element-1 (LINE-1) and short interspersed nuclear elements (SINEs) have been suggested as key developmental events of cancer^3, 4^. Global DNA methylation has been examined in surrogate tissues, including leukocyte DNA^5^. Overall methylation levels reflect overall alterations in gene expression. Low levels of leukocyte global DNA methylation have been associated with increased risks for various cancers, including head and neck squamous cell carcinoma, bladder, breast, gastric, and colorectal adenoma in adults^6^. Our recent research has shown that leukocyte DNA methylation alterations (globally or locally) are associated with obesity, hypertension, and vitamin D deficiency in adolescents^7–11^.

Telomere shortening has been proposed as a mechanism for decreasing chromosomal stability, which increases risks for chronic diseases, cancer, cardiovascular disease, and overall mortality^12–15^. Mutations in telomerase and telomere components lead to a broad spectrum of disease that has presentations in adults and children. The syndromes of short telomeres, such as Dyskeratosis Congenita, is marked by vascular and degenerative components as well as by cancer predisposition^16, 17^. We have previously shown that diet and physical activity influence telomere dynamics in adolescents^18–20^.

Both leukocyte global DNA methylation and telomeres are involved in aging processes and disease vulnerability. Emerging studies have suggested that global DNA methylation may help regulate the variability of telomere length (LTL) in adults^21, 22^. LINE-1 DNA hypomethylation, a surrogate for global DNA hypomethylation, may decrease the methylation of subtelomeric regions, which are associated with short telomere length^21^. Moreover, DNA methylation levels in multiple subtelomeric and imprinted loci were associated with leukocyte telomere lengths^22^. However, the relationships between epigenetics and telomeres remain unknown in youth. The present study, for the first time, tested the hypothesis that lower levels of %5 mC would be associated with shorter LTL.

## Results

The general characteristics, levels of %5 mC, and LTL of our subjects were presented by gender and ethnicity in Table 1.There were a total of 542 participants in our dataset (44.8% African Americans; 55.2% females) who qualified for our analysis. Females when compared to males were significantly shorter and lighter and had higher levels of %5 mC (*p* < 0.01). Additionally as previously reported, females had fewer minutes of vigorous physical activity (PA)^18^ (*p* < 0.05). Likewise as previously reported, African Americans were heavier and had longer LTL than Whites^11, 18^.

**Table 1:** General characteristics of study participants by gender and ethnicity.

Variables Assessed	Females	Males	African-Americans	Whites	P-Value	
					Gender	Ethnicity
Total	299	243	243	299	542	542
Age	16.13 ± 1.21	16.06 ± 1.21	16.13 ± 1.23	16.06 ± 1.21	0.65	0.53
Height (m)	1.63 ± 5.97	1.74 ± 7.58	1.68 ± 9.03	1.69 ± 8.54	< 0.01	0.10
Weight (kg)	61.40 ± 14.09	68.90 ± 14.61	67.41 ± 15.77	62.68 ± 13.63	< 0.01	< 0.01
BMI (kg/m^2)	23.08 ± 5.21	22.54 ± 4.17	24.01 ± 5.38	21.89 ± 3.98	0.19	< 0.01
Vigorous Physical Activity (minutes/day)	1.96 ± 2.95	5.43 ± 7.38	3.77 ± 6.43	3.34 ± 5.03	< 0.01	0.41
Tanner Stage	4.38 ± 0.69	4.31 ± 0.78	4.43 ± 0.70	4.29 ± 0.75	0.26	< 0.02
C5 Methylation (%5 mC)	0.46 ± 0.22	0.42 ± 0.24	0.42 ± 0.23	0.45 ± 0.23	< 0.01	0.30
Telomere Length (T/S ratio)	1.31 ± 0.21	1.28 ± 0.24	1.32 ± 0.21	1.27 ± 0.23	0.19	< 0.01

**Figure 1:**
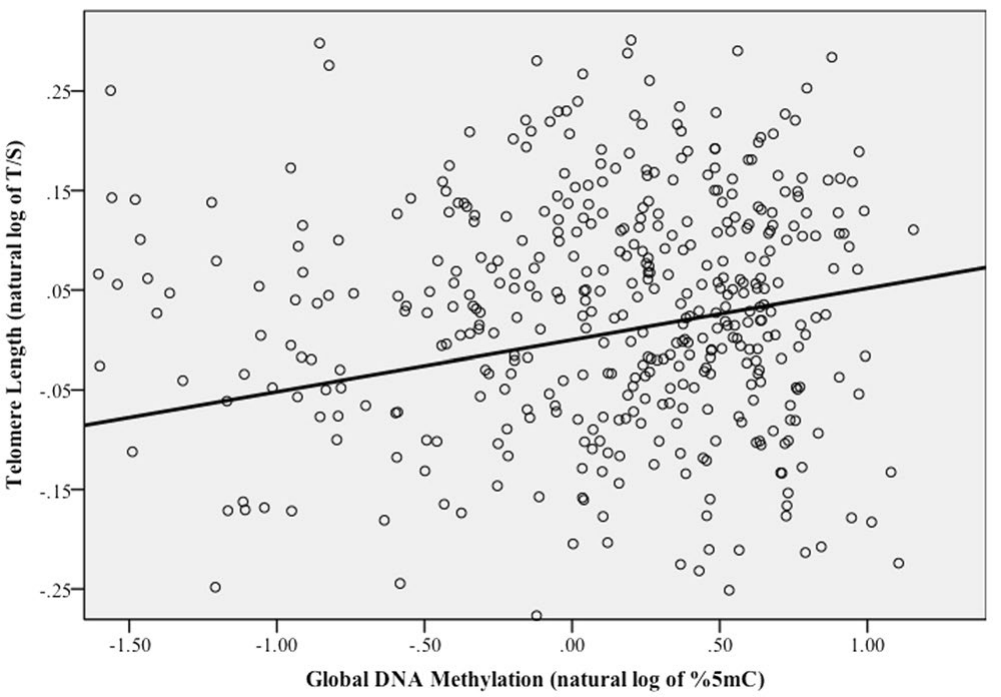
Partial regression plot of the model between Global DNA Methylation (natural log of %5 mC) and Telomere Length (natural log of T/S) for the overall population.

In the whole sample, multiple linear regression model adjusted for age, gender, ethnicity, Tanner stage, BMI, and batch effect was significant (*p* < 0.01, Table 2). Unadjusted linear regressions with batch-effect of %5 mC as co-variate identified the positive association between %5 mC and LTL (Table 3-Unadjusted). Adjusted %5 mC also was positively associated with LTL (adjusted β = 0.15, *p* < 0.01) (Table 3, Adjusted Model 1) The significance remained after further adjusted for physical activity (adjusted β = 0.17, *p* < 0.01, Table 3, Adjusted Model 2, Fig. 1) and %5 mC accounted for 5.0% of the variation in LTL (adjusted R^2^ = 0.05).

**Table 2:** Fit of the model for overall population.

Overall fit	Sum of Squares	df	Mean Square	F	P-Value
Regression	1.441	7	0.206	4.185	0.000
Residual	26.270	534	0.049		
Total	27.711	541			

Adjusting for age, gender, ethnicity, BMI, Tanner stage, batch effect.

**Table 3:** Linear regression model for overall population.

Model	Unstandardized Coefficients		Standardized Coefficients	P-Value	95.0% Confidence Interval for B	
	B	Std. Error	Beta (β)		Lower Bound	Upper Bound
Unadjusted	0.043	0.012	0.147	0.000	0.019	0.067
Adjusted Model 1*	0.045	0.012	0.154	0.000	0.020	0.069
Adjusted Model 2**	0.052	0.014	0.170	0.000	0.025	0.079

^*^Adjusting for Age, Gender, Ethnicity, BMI, Tanner Stage, Batch Effect.

**Adjusting for Age, Gender, Ethnicity, BMI, Tanner Stage, Batch Effect, Physical Activity.

A significant interaction with gender was identified (*p < *0.01). There was a significant association between %5 mC and LTL in females (all *ps* < 0.01), but not in males (Table 4). Further sensitivity analyses by race revealed similar associations in African Americans and whites (all *ps* < 0.03, Table 5). These associations were maintained after further adjusting for physical activity in these different groups (Tables 4 and 5, Adjusted Model 2).

**Table 4:** Linear regression model partitioned by gender.

Model with gender stratified results		Unstandardized Coefficients	Standardized Coefficients		P-Value	95.0% Confidence Interval for B	
		B	Std. Error	Beta (β)		Lower Bound	Upper Bound
Male	Unadjusted	0.005	0.016	0.021	0.734	−0.025	0.036
	Adjusted Model 1*	0.011	0.016	0.044	0.497	−0.020	0.042
	Adjusted Model 2**	0.014	0.018	0.054	0.432	−0.021	0.049
Female	Unadjusted	0.091	0.019	0.265	0.000	0.054	0.129
	Adjusted Model 1*	0.089	0.019	0.261	0.000	0.051	0.127
	Adjusted Model 2**	0.094	0.021	0.268	0.000	0.052	0.136

^*^Adjusting for Age, Gender, Ethnicity, BMI, Tanner Stage, Batch Effect.

**Adjusting for Age, Gender, Ethnicity, BMI, Tanner Stage, Batch Effect, Physical Activity.

**Table 5:** Linear regression model partitioned by ethnicity.

Model with ethnicity stratified results		Unstandardized Coefficients	Standardized Coefficients		P-Value	95.0% Confidence Interval for B	
		B	Std. Error	Beta (β)		Lower Bound	Upper Bound
White	Unadjusted	0.052	0.018	0.163	0.004	0.016	0.087
	Adjusted Model 1*	0.052	0.019	0.163	0.006	0.015	0.089
	Adjusted Model 2**	0.061	0.021	0.179	0.004	0.019	0.102
African American	Unadjusted	0.035	0.015	0.145	0.022	0.005	0.066
	Adjusted Model 1*	0.035	0.015	0.147	0.024	0.005	0.066
	Adjusted Model 2**	0.039	0.017	0.162	0.019	0.006	0.072

^*^Adjusting for Age, Gender, Ethnicity, BMI, Tanner Stage, Batch Effect.

**Adjusting for Age, Gender, Ethnicity, BMI, Tanner Stage, Batch Effect, Physical Activity.

## Discussion

In our population of African American and White high school students, living in the Southeastern United States, we found that leukocyte DNA methylation levels were associated with LTL. Evidence suggests that epigenetics may influence telomere length maintenance and that both global DNA methylation and telomere length are inversely associated with aging and chronic disease risk^23–28^.

A mice study first described a role for mammalian DNA methyltransferases in telomere length control, which demonstrated a previously unappreciated role for DNA methylation in telomere maintenance^29^. Ng *et al*. investigating the peripheral blood mononuclear cells of human adults, found that an increase in global DNA methylation at the proximal subtelomeric regions was associated with an increase in telomerase activity, known to be involved in telomere maintenance^30^. Yehezkel *et al*. found that global DNA hypomethylation of subtelomeric regions in lymphoblastoid and fibroblast cells of patients diagnosed with immunodeficiency was associated with shorter telomeres^31^.

Our finding that lower levels of global DNA methylation were associated with shorter LTL in healthy adolescents was supported by several studies in healthy adults and patients. The Alu and LINE-1 DNA hypomethylation found in biliary atresia patients were associated with shorter telomere length^4^. The Harvard Boilermakers Longitudinal Study was a prospective open-cohort study with 400 active union members, participating in a two-year follow-up study. They showed that LINE-1 methylation levels were associated with LTL at the baseline and longitudinally^21^. In an epigenome wide study using Illumina 450 K array, methylation levels at 65 gene promoters in subtelomeric and imprinted loci were associated with LTL^22^. Additionally, the only pediatric research on global DNA methylation and telomere length identified a positive relationship between LINE-1 DNA hypomethylation and telomere shortening in youth with Wilms Tumor^32^.

Our sensitivity analyses by ethnicity and gender revealed that the positive associations between %5 mC and LTL were observed across the 4 groups with 3 groups achieving statistical significance except the male group. To the best of our knowledge, the present study is the largest study conducted to date. The studies cited in the previous paragraph with sample sizes ranging from 20–228 have not looked at and reported the ethnicity and gender influence on the association. Despite the lack of significance in the male group, the same trend existed. Further studies are warranted to validate these findings.

As mentioned in our results, we observed that females had higher levels of global DNA methylation as compared to males. Zhu *et al*. reported that females had higher Alu methylation, although the majority of studies done on LINE-1 DNA methylation have found that males had higher LINE-1 DNA methylation levels^6, 33–36^. Hall *et al*. investigated gender differences in genome-wide DNA methylation pattern in human pancreatic islets using Illumina 450 beadchip kit and discovered that females had higher genome-wide methylation level on X chromosome, while the autosomes did not display gender difference in DNA methylation level^37^. The methylflash kit we used was designed to detect DNA methylation at any locus across genome including all repetitive elements using specific detection antibodies; therefore, our %5 mC results represented the global level of DNA methylation. Gender differences in global DNA methylation require further investigation.

There were several notable strengths in our study. First, we recruited a relatively large and apparently healthy adolescent population with near equal distributions by gender and ethnicity. Second, we recruited adolescents with a narrow range of ages, minimizing the confounding effects of maturation, disease development, and chronological aging on %5 mC and LTL. Limitations of our study should also be recognized. First, because this was a cross-sectional study, the associations did not prove causality. Second, because tissue samples were not collected in those healthy adolescents, available buffy coat samples were analyzed. However, LTL and leukocyte DNA methylation are commonly used in epidemiological studies, which have shown to be highly correlated with tissue levels^38, 39^.

## Conclusion

In a healthy adolescent population, for the first time, we observe a positive association between leukocyte global DNA methylation and telomere length. Global DNA hypomethylation may be an underlying mechanism that affects telomere length detrimentally. Because as early as birth global DNA methylation and telomere length both play a role in growth and development, cellular aging, chromosomal stability, and disease vulnerability, our study contributes to the understanding of the molecular and genetic mechanism underlying chronic disease risk. Further longitudinal studies are warranted to study the influence of global DNA methylation on telomere length over time.

## Subjects and Methods

### Participants

A cross-sectional study was conducted in a cohort of apparently healthy African American and White adolescents aged 14 to 18 years old (n = 542: 44.8% African Americans; 55.2% females). Participants were recruited, from local public high schools in the Augusta, Georgia area (located in Southeast US), to take part in the Lifestyle, Adiposity, and Cardiovascular Health in Youth (LACHY) study. Participants were excluded with the following self-reported conditions: taking current medications or diagnosed with chronic medical conditions that could affect growth, maturation, physical activity, nutritional status, or metabolism.

### Ethics Statement

The Institutional Review Board at the Medical College of Georgia, Augusta University approved this project (Augusta, GA, USA, protocol #622505). Flyers, approved by school superintendents and principals, were distributed to all high schools. Written informed consent was obtained from all adult subjects and from parents/guardians of those younger than 18 years old. All methods were performed in accordance with the relevant guidelines and regulations. Each participant was assigned a unique subject number. All data were anonymized and de-identified prior to analysis.

### Anthropometry and sexual maturation stage

Height and weight were obtained according to standard procedures, using a wall-mounted stadiometer (Tanita Corporation of American, Arlington Heights, IL) and calibrated electronic scale (model CN2OL; Cardinal Detecto, Webb City, MO). Prior to testing each week, the electronic scale was checked for accuracy using known weights. BMI was calculated as weight (kg) divided by height (m^2^).

Sexual development of the participants was measured by a five-stage scale, ranging from 1 (prepubertal) to 5 (fully mature) as described by Tanner^40^. Using a gender-specific questionnaire, the subjects reported their sexual maturation stage by comparing their own physical development to the five stages in standard sets of diagrams. A parent or research coordinator then reviewed the results with the youth to make sure they understood the questionnaire. When an individual reported discordant stages of pubic hair and breast or genital development, the higher of the two stages was used.

### Physical Activity

The total daily minutes spent for moderate and vigorous physical activities (PA) were assessed using MTI Actigraph monitors (model 7164; MTI Health Services, Fort Walton Beach, FL), uniaxial accelerometers that measure vertical acceleration and deceleration. With epoch length set at 1 min and expressed as counts per minute, the accelerometers started recording when the subject left our institute after the first testing day. The subjects were instructed to 1) wear the monitor for a period of 7 days, 2) remove it for sleep, bathing, and any activity that may cause harm to either the monitor or another person (e.g. during contact sports), and 3) bring the monitor back to us after 1 week. Data from days 1 and 7 were discarded because a full day of information was not available. Movement counts were converted to average minutes per day spent in moderate (3–6 metabolic equivalents) and vigorous ( > 6 metabolic equivalents) PA by the software accompanying the device.

### Global DNA Methylation Assessment

Genomic DNA was extracted from stored buffy coat samples using Qiagen® QiAamp® DNA Mini Kit (Qiagen Inc. Valencia, CA). DNA samples with OD 260/280 and 260/230 greater than 1.8 were used for global DNA methylation measurement. Global DNA methylation level was quantified in 100 ng genomic DNA using leukocyte DNA with the MethylFlashTM Methylated DNA Quantification kit (Epigentek, Farmingdale, NY) following manufacturer’s instruction. Briefly, the methylated DNA was detected using capture and detection antibodies to 5-methylcytosine (5 mC) and then quantified colorimetrically by reading absorbance at 450 nm using Bio-Tek PowerWave HT Microplate Spectrophotometer (biotek, Winooski, VT). The amount of methylated DNA is proportional to the OD intensity measured. Relative quantification was used to calculate percentage of 5-mC (%5 mC) in total leukocyte DNA following the manufacturer’s instructions. Each sample was run in duplicate.

### Measurement of Leukocyte Telomere Length (LTL)

Mean LTL was determined from leukocyte DNA by a modified quantitative PCR-based assay as previously described^18, 41^. The relative ratio of telomere repeat copy number (T) to single-copy gene copy number (36B4 gene, encoding ribosomal phosphoprotein, located on chromosome 12, S) was determined using a *7500 Fast real-time PCR System* (Applied Biosystems, Foster City, CA, USA). Samples were done in triplicate. Threshold values (Ct) were obtained by averaging the triplicates. Each 96-well plate contained a five-point standard curve using the same control genomic DNA from 3 to 48 ng to control the day-to-day variations. Standard curves with linearity R2 > 0.98 were accepted. Telomere PCRs and 36B4 PCRs were performed on separate plates, with the same sample well position. T/S ratio was calculated as: the amount of telomeric DNA (T) divided by the amount of single-copy control gene DNA (S). The intra-plate and inter-plate coefficients of variation for the T/S ratio were 5.6% and 6.8%, respectively^18^.

### Statistical Analysis

Descriptive statistics for raw variables are presented as mean ± standard deviation (see Table 1). Prior to analysis, the normal distributions and homogeneity of all variables were checked using histograms, Shapiro-Wilks *W* test and Levene’s test for equality of variances. Since %5 mC and LTL deviated from normality significantly, natural log transformations were applied. Ethnicity and gender differences were checked for age, gender, BMI, PA, Tanner Stage, levels of %5 mC, and LTL, using independent samples two-tailed *t*-tests.

Multiple linear regression models were used to estimate the associations between %5 mC as the main predictor and LTL as the outcome. The first model utilized unadjusted linear regressions with batch effect of %5 mC as a co-variate to analyze the overall population. The second model used linear regressions, adjusted for age, gender, ethnicity, Tanner stage, BMI, and batch effect as covariates, to analyze the overall population. Finally, the third model, which further adjusted for physical activity, was analyzed for the overall population. The overall fit of the model and a partial regression plot with %5 mC as the independent variable and LTL as the dependent variable were provided. The interactions of %5 mC with ethnicity and gender were tested in separate models. All three linear regression models were repeated after stratifying the data by gender and ethnicity separately.

All statistical analyses were performed via SPSS –IBM Software (version 24.0 SPSS Inc., Chicago, IL, USA) with the significance level set at *α* = 0.05.
